# Flight training and alterations in cortical characteristics

**DOI:** 10.3389/fnhum.2025.1672307

**Published:** 2025-09-16

**Authors:** Xi Chen, Qi Chu, Hongming Wang, Xi Tan, Mingjun Duan, Cheng Luo

**Affiliations:** ^1^The Clinical Hospital of Chengdu Brain Science Institute, MOE Key Lab for Neuroinformation, University of Electronic Science and Technology of China, Chengdu, China; ^2^Institute of Flight Technology, Civil Aviation Flight University of China, Guanghan, China

**Keywords:** cortical characteristics, flight cadets, flight training, gray matter thickness, cortical surface area, sulcal depth

## Abstract

**Background:**

Piloting is a highly specialized profession. The purpose of this study was to investigate the effects of longitudinal flight training on the cortical characteristics of the brain.

**Methods:**

Magnetic resonance imaging of 25 flight cadets and 24 controls was performed twice over a 2-year period. The gray matter thickness, cortical surface area and sulcal depth of the two groups were compared. The relationships between altered cortical characteristics and flight training days were investigated through correlation analysis.

**Results:**

The results indicated that flight training was associated with certain parameters of brain structure. Specifically, in the left rostral anterior cingulate area, the flight group displayed prominent gyri, whereas the control group exhibited sulci. The structural changes in these two groups followed completely opposite trends.

**Conclusion:**

In conclusion, the affected areas are primarily concentrated in brain regions associated with multisensory integration and multitasking, which may reflect neural adaptations induced by flight training.

## 1 Introduction

The flight environment is dynamic and complex. Modern aircraft are highly automated and require fewer human interventions than before. The pilot’s role has shifted to that of a monitor and decision-maker who oversees automated systems. Due to both the high degree of automation and the complexity of data generated during flight operations, aviation systems have become increasingly difficult for laypeople to comprehend. Consequently, piloting has evolved into a highly specialized profession that demands rigorous selection criteria combined with years of professional training. Previous studies have demonstrated that pilots have some unique brain functional and structural characteristics (Adamson et al., 2012; Ahamed et al., 2014). Similarly, our team identified significant differences in brain function between pilots and the general population. A critical question remains: do these neurobiological differences originate from pre-screening or develop through post-training processes? To address this question, longitudinal studies tracking cadets are essential. Longitudinal tracking studies allow for the observation of changes in brain structure within the same group of participants over the course of training, thereby eliminating the confounding effects of individual differences. By establishing a control group, the influence of time-related factors can also be controlled. As a result, the brain structural changes specifically attributable to flight training can be accurately identified. Should these disparities be attributable to training, this would suggest neural plasticity that could inform optimized training protocols. Conversely, if such differences predate training, they might represent stable traits suitable for objective selection criteria in aviation recruitment.

Humans respond to new challenges throughout their lives. The connectivity of the human brain is only partially determined by genetics and early environmental factors, and can be modified through experiences (Pantev and Herholz, 2011). Studies have demonstrated that both cognitive training and physical exercise can induce structural neuroplastic changes in the brain (Gaser and Schlaug, 2003; Draganski et al., 2004; Draganski et al., 2006; Boyke et al., 2008). Our research team has recently discovered that flight training induces both functional reorganization in brain networks and microstructural alterations in white matter fiber tracts (Chen et al., 2020, 2023, 2024). What is the relationship between flight experience and the structural organization of gray matter in the human brain?

Quantitative approaches using brain magnetic resonance imaging (MRI) have been used to characterize distinctive brain alterations caused by flight experience. The Stanford/VA Aviation Study examined the influence of specialized training and skill levels on brain structure, revealing that a larger parietal lobe in expert pilots correlated with better performance in flight simulators (Adamson et al., 2010). A large-sample study targeting on mature pilots demonstrated that structural alterations of cortical thickness were more pronounced in pilots compared to non-pilots (Cao et al., 2019). Subsequent VBM studies further revealed that pilots exhibited increased gray matter volume in the lingual gyrus, fusiform gyrus, inferior frontal gyrus, supramarginal gyrus, cuneus, and postcentral gyrus compared to the control group (Qiu et al., 2021; Xu et al., 2023). These studies suggest that there are indeed differences in brain structure between pilots and the general population, the majority of the brain regions exhibiting these changes are associated with sensorimotor functions. Our previous longitudinal studies have also revealed that flight training can induce changes in the activation levels of brain functional networks and also bring about subtle modifications in white matter structure (Chen et al., 2020, 2024). Thus, we hypothesize that flight training will result in measurable changes in cortical morphometry, especially in multisensory and motor regions.

Cortical characteristics, such as cortical thickness, surface area, and sulcal depth, have been shown to correlate with aging (Jin et al., 2018), cognitive performance (Natu et al., 2021; Yao et al., 2023) and pathological changes (Lyu et al., 2018; Nickel et al., 2019). These morphological features reflect the structural organization of the cerebral cortex and may provide precise biomarkers for understanding neuroplastic mechanisms associated with flight training. The present study aimed to investigate flight training-induced alterations in cortical morphology. To achieve this, we implemented a fully data-driven analytical approach to detect structural changes in cerebral cortex architecture, thereby establishing a methodological framework for future research in this field.

## 2 Materials and methods

### 2.1 Participants and study design

After excluding individuals with a history of neurological diseases, traumatic brain injury, or substance-related disorders, we recruited 49 subjects (24 college students and 25 flight cadets) from the Civil Aviation Flight University of China (CAFUC), assigning them to the control group and flight group, respectively. No participants dropped out during the study. The students in the control group were majoring in cognitive psychology and engine maintenance, both of which fall within the broader domain of science and engineering disciplines. Consequently, the difference in cognitive load between the course content of the control group and the theoretical components of the flight cadets’ curriculum is unlikely to constitute a significant confounding variable.

Data was collected at two time points during the study. The ab initio training program at CAFUC comprises 2 years of ground-based theoretical coursework followed by approximately 2 years of practical flight training. In contrast, the control group students completed 2 years of purely theoretical studies with no exposure to flight operations. Consequently, no differences in training content existed between the two groups during the first 2 years.

The first data collection occurred in 2019 when all 49 participants were freshmen (approximately 6 months after enrollment), during which T1-weighted magnetic resonance imaging (MRI) structural brain scans were acquired. The second data collection took place in 2022, 31–41 months after the initial assessment (The recruitment period for the entire experimental subjects was from January 1, 2019 to June 30, 2022.). During this interval: The control group completed 3 years of standard academic coursework. The flight group underwent 40 h of simulated flight training and 200 h actual aircraft flight with variable durations of actual flight training, in addition to completing their remaining two-year theoretical curriculum. Aircraft types included the C172R and DA-42. The training is a continuous process that includes various levels of difficulty stages. All the flight cadets received the same flight training, but since each person’s progress was different, the time it took for them to complete all the training varied. At the second assessment, T1-weighted MRI data were collected again, and the cumulative flight training hours for each cadet were documented.

The study protocol was approved by the Ethics Committee of the University of Electronic Science and Technology of China (No. 1420200408–07). All the participants signed the informed consent form for participating in the experiment.

### 2.2 MRI data acquisition

T1-weighted images of all subjects were acquired using a 3-T MRI (DISCOVERY MR 750, GE Healthcare, USA) scanner at the University of Electronic Science and Technology of China. T1-spoiled gradient recalled echo pulse sequences were used for data acquisition (scan acquisition direction, axial; repetition time, 5.976 ms; echo time, 1.976 ms; matrix, 256 × 256; flip angle, 9°; field of view, 25.6 cm × 25.6 cm × 15.4 cm; slice thickness, 1 mm; number of slices, 154).

### 2.3 MRI data processing

The FreeSurfer software suite (v7.4.1)^1^ was utilized to process T1-weighted MRI data for extracting three neuroanatomical parameters: cortical thickness, cortical surface area, and sulcal depth. These morphometric indices represent the most widely adopted measurements in cerebral cortex structural research. The processing pipeline comprised four sequential stages:

(1) Preprocessing: segmentation of each subject’s T1-weighted image, encompassing non-brain tissue removal (Segonne et al., 2004), intensity normalization (Sled et al., 1998), and tissue segmentation (Fischl et al., 2004). Subdivision of the gray matter/white matter boundaries, triangulation, and topological correction were performed to obtain precise gray matter/white matter surfaces (Segonne et al., 2007). (2) Gray matter thickness: cortical thickness was calculated by determining the shortest distance between the gray matter/white matter surface and each vertex on the pial surface (Fischl and Dale, 2000). (3) Cortical surface area: using a triangulated precision surface, the cortical surface area index was computed by summing the vertex-based surface areas. (4) Sulcal depth: FreeSurfer’s automatic algorithm facilitated the determination of sulcal depth for each subject. Positive values represent the depths of the sulci, whereas negative values indicate the heights of the gyri.

### 2.4 Statistical analyses

Statistical analyses of demographic parameters were conducted using SPSS software (version 26.0). A two-tailed *t*-test was used to compare age between flying cadets and ground controls, and the chi-square test was employed to compare handedness ratios.

FreeSurfer’s Qdec tool was utilized for MRI data analysis. The Qdec table was constructed based on participants’ demographic and clinical information. All cortical metrics were integrated, smoothed with a Gaussian kernel (FWHM = 10 mm) (Marie et al., 2016; Yun et al., 2020), and analyzed using a linear mixed model to perform a 2 × 2 ANOVA (time × group interaction). Statistical significance was defined as a vertex-wise threshold of *p* < 0.001 (uncorrected). Cortical parcellation followed the Desikan-Killiany (DK) atlas. Regions showing significant interaction effects underwent *post-hoc* analysis (Bonferroni-corrected *p* < 0.05).

Spearman’s correlation coefficients were calculated to assess relationships between flight training duration and cortical metrics with significant group differences. All tests were two-sided, with the significance level set at *p* < 0.05.

## 3 Result

### 3.1 The demographic profile of the participants

No significant differences were observed between the two groups in sex, age, or dominant hand. Table 1 presents the demographic data of both groups. The flight training duration refers to the number of days required for cadets to complete the entire flight training program.

**Table 1:** Demographic profile between the two groups.

	Flying cadet (*n* = 25)	Ground control (*n* = 24)	*t*-value (Chi-square test)	*p*-value
Age (years)^a^	18.84 ± 0.69	18.88 ± 0.85	−0.159^c^	0.88
Sex (male)	100%	100%		
Handedness (right)^b^	96%	92%	0.40^d^	0.53
Flight training duration (days)	376.82 ± 56.29			

Data are presented as means ± standard deviations.

a The age of subjects in 2019.

b The proportion of right-handed people among the subjects.

c Two-tailed *t*-tests.

d Chi-square tests.

### 3.2 Interaction effect

The interaction effect (group vs. time) was shown in the cortical thickness (left superior-parietal area, left supramarginal, right entorhinal and right postcentral area), cortical surface area (left entorhinal, left postcentral, left paracentral, left superior-frontal, right inferior-parietal, right precentral area) and sulcal depth (left lateral-occipital and rostral anterior cingulate area) (Table 2). This interaction indicated that the two groups exhibited distinct morphological change trajectories between the two time points.

**Table 2:** The regions with interaction effects (vertex, *p* < 0.001).

	Brain area	Peak vertex (MNI)			−log10P	Cluster size (mm^2^)
		X	Y	Z		
Cortical thickness	Superiorparietal_l	−19.6	−64.5	37.9	4.2467	77.75
	Supramarginal_l	−38.5	−38.3	16.5	3.8428	16.17
	Entorhinal_r	20.8	−11.3	−28.2	3.7254	34.96
	Postcentral_r	61	−5.4	10.3	3.5061	12.6
Cortical surface area	Entorhinal_l	−30	−16.2	−30.2	3.9382	40.61
	Postcentral_l	−31.3	−30	49.9	5.1446	38.94
	Paracentral_l	−5.2	−28.4	63.9	3.5932	22.53
	Superiorfrontal_l	−11.1	32.7	28.4	3.2299	12.21
	Inferiorparietal_r	37.5	−83.4	15.6	4.8793	179.13
	Inferiortemporal_r	46.3	−19	−27.1	3.5031	37.01
	Precentral_r	54.2	−4.3	20.5	4.2743	22.65
Sulcal depth	Lateraloccipital_l	−27.6	−94.9	−8.1	3.2195	31.54
	Rostralanteriorcingulate_l	−1.8	32.2	6.6	3.6463	22.72

l, left hemisphere; r, right hemisphere.

### 3.3 *Post-hoc* analysis

**Figure 1:**
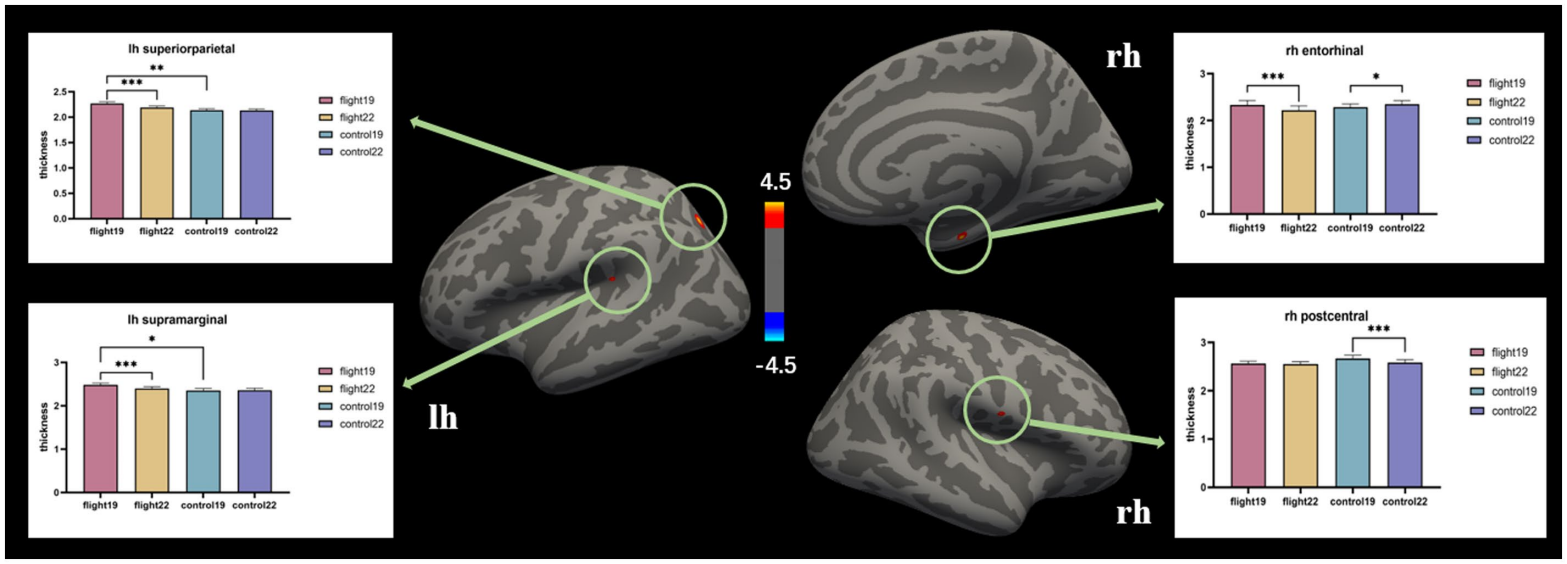
The brain regions with significant interaction effects on the index of cortical thickness and the results of *post-hoc* tests. lh, Left hemisphere; rh, right hemisphere.

Cortical thickness. At baseline, the left hemisphere’s superior parietal and supramarginal areas exhibited greater cortical thickness in the flight group compared to the control group. After about 2 years, however, these regions showed significant cortical thinning in the flight group. In the right hemisphere, the entorhinal cortex demonstrated contrasting trajectories: it was thicker in the control group at follow-up (second visit), whereas the flight group displayed cortical thinning in this region during the same period. Similarly, the right postcentral area exhibited significant thinning in the control group at follow-up, while the flight group showed minimal structural changes in this region between baseline and follow-up assessments (Table 3; Figure 1).

**Table 3:** *Post-hoc* test results of significant regions of interaction in cortical thickness (*p* < 0.05, Bonferroni).

Brain area	Comparison	*p* value	Mean difference	Cohen’s d
Superiorparietal_l	Flight19-control19	0.0025	0.1354	2.1430
Superiorparietal_l	Flight22-control22	0.1767	0.0574	0.9080
Superiorparietal_l	Flight22-flight19	0.0000	−0.0823	1.3030
Superiorparietal_l	Control22-control19	0.7405	−0.0043	0.0690
Supramarginal_l	Flight19-control19	0.0424	0.1289	1.6960
Supramarginal_l	Flight22-control22	0.5385	0.0402	0.5290
Supramarginal_l	Flight22-flight19	0.0000	−0.0860	1.1320
Supramarginal_l	Control22-control19	0.8631	0.0027	−0.0360
Entorhinal_r	Flight19-control19	0.6758	0.0497	0.3180
Entorhinal_r	Flight22-control22	0.2998	−0.1292	−0.8260
Entorhinal_r	Flight22-flight19	0.0008	−0.1135	0.7250
Entorhinal_r	Control22-control19	0.0482	0.0654	−0.4180
Postcentral_r	Flight19-control19	0.1787	−0.1062	−1.5260
Postcentral_r	Flight22-control22	0.7084	−0.0289	−0.4160
Postcentral_r	Flight22-flight19	0.4189	−0.0115	0.1650
Postcentral_r	Control22-control19	0.0000	−0.0888	1.2750

l, left hemisphere; r, right hemisphere.

**Figure 2:**
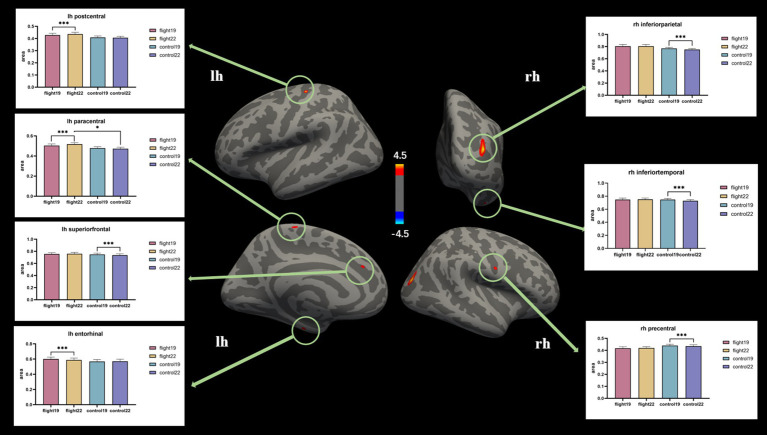
The brain regions with significant interaction effects on the index of cortical surface area and the results of *post-hoc* tests. lh, Left hemisphere; rh, right hemisphere.

Cortical surface area. The cortical surface area of the left postcentral gyrus and left paracentral lobule in the flight group showed a significant increase at the second visit (2022). In contrast, the cortical surface area of the left superior frontal gyrus, right inferior parietal lobule, right inferior temporal gyrus, and right precentral gyrus in the control group exhibited a significant decrease at the second visit (Table 4; Figure 2).

**Table 4:** *Post-hoc* test results of significant regions of interaction in cortical surface area (*p* < 0.05, Bonferroni).

Brain area	Comparison	*p* value	Mean difference	Cohen’s d
Entorhinal_l	Flight19-control19	0.3687	0.0332	2.5630
Entorhinal_l	Flight22-control22	0.6512	0.0168	1.2980
Entorhinal_l	Flight22-flight19	0.0000	−0.0134	1.0330
Entorhinal_l	Control22-control19	0.2659	0.0030	−0.2320
Postcentral_l	Flight19-control19	0.2940	0.0194	2.6590
Postcentral_l	Flight22-control22	0.1098	0.0297	4.0550
Postcentral_l	Flight22-flight19	0.0000	0.0080	−1.0990
Postcentral_l	Control22-control19	0.1559	−0.0022	0.2970
Paracentral_l	Flight19-control19	0.2240	0.0257	1.5210
Paracentral_l	Flight22-control22	0.0407	0.0449	2.6550
Paracentral_l	Flight22-flight19	0.0002	0.0138	−0.8140
Paracentral_l	Control22-control19	0.1278	−0.0054	0.3200
Superiorfrontal_l	Flight19-control19	0.7986	0.0080	0.5990
Superiorfrontal_l	Flight22-control22	0.4725	0.0223	1.6660
Superiorfrontal_l	Flight22-flight19	0.2005	0.0035	−0.2620
Superiorfrontal_l	Control22-control19	0.0003	−0.0108	0.8050
Inferiorparietal_r	Flight19-control19	0.2973	0.0361	3.2040
Inferiorparietal_r	Flight22-control22	0.1214	0.0541	4.7920
Inferiorparietal_r	Flight22-flight19	0.8635	−0.0004	0.0350
Inferiorparietal_r	Control22-control19	0.0000	−0.0183	1.6230
Inferiortemporal_r	Flight19-control19	0.9829	0.0006	0.0330
Inferiortemporal_r	Flight22-control22	0.4417	0.0214	1.1910
Inferiortemporal_r	Flight22-flight19	0.6236	0.0018	−0.1000
Inferiortemporal_r	Control22-control19	0.0000	−0.0190	1.0580
Precentral_r	Flight19-control19	0.1858	−0.0223	−4.2400
Precentral_r	Flight22-control22	0.3434	−0.0155	−2.9520
Precentral_r	Flight22-flight19	0.0950	0.0018	−0.3440
Precentral_r	Control22-control19	0.0000	−0.0050	0.9430

l, left hemisphere; r, right hemisphere.

**Figure 3:**
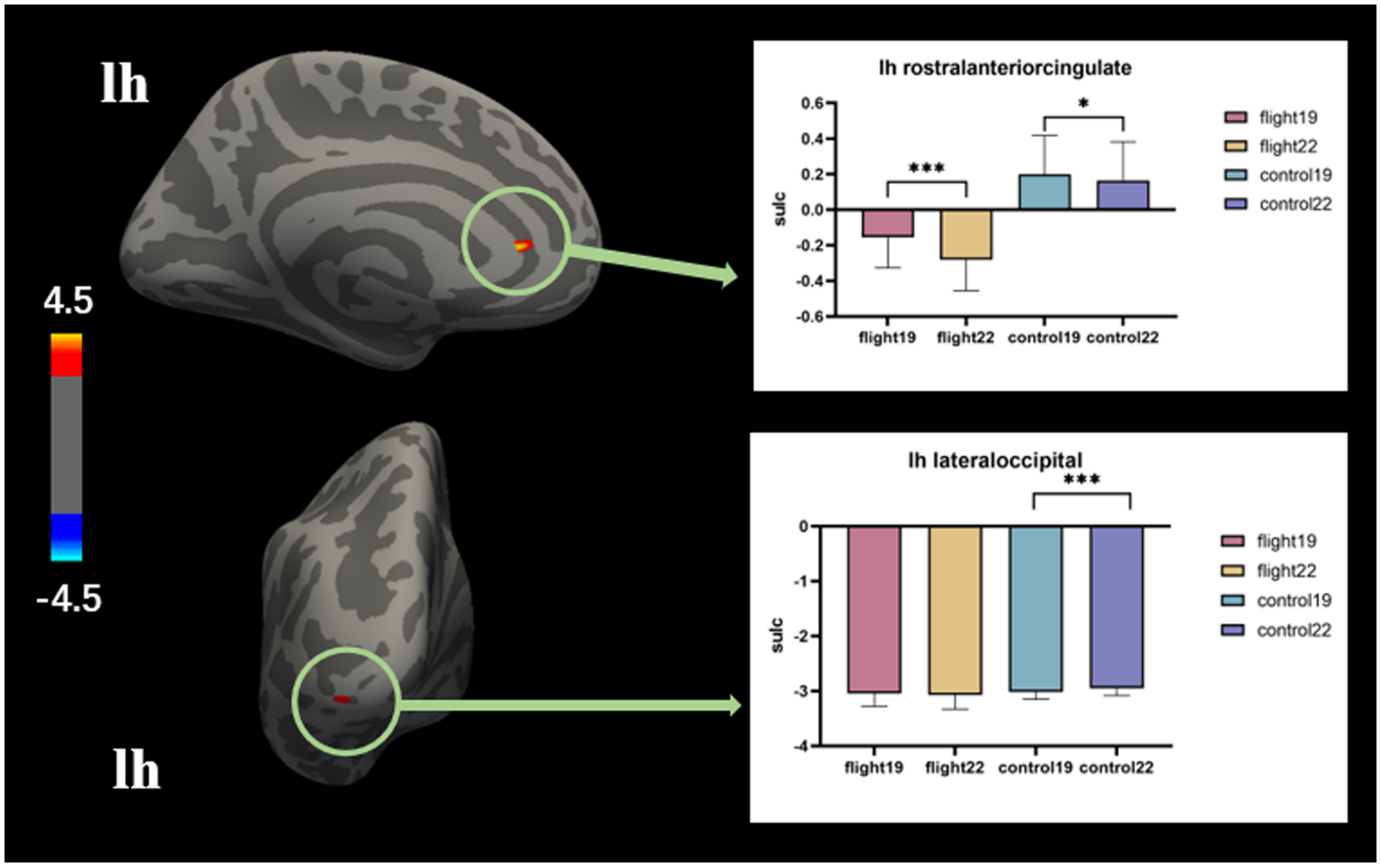
The brain regions with significant interaction effects on the index of sulcal depth and the results of *post-hoc* tests. lh, Left hemisphere.

Sulcal depth. The negative values correspond to gyri, whereas the positive values represent sulci. Sharper curvature is associated with higher absolute values. In the left rostral anterior cingulate region, the flight group exhibited gyri morphology, while the control group displayed sulci. The discrepancies observed during the initial measurement demonstrated an increase in magnitude during the subsequent measurement. (Table 5; Figure 3).

**Table 5:** *Post-hoc* test results of significant regions of interaction in sulcal depth (*p* < 0.05, Bonferroni).

Brain area	Comparison	*p* value	Mean difference	Cohen’s d
Lateraloccipital_l	Flight19-control19	0.9381	−0.0222	−0.2280
Lateraloccipital_l	Flight22-control22	0.6715	−0.1251	−1.2870
Lateraloccipital_l	Flight22-flight19	0.1078	−0.0322	0.3310
Lateraloccipital_l	Control22-control19	0.0010	0.0707	−0.7270
Rostralanteriorcingulate_l	Flight19-control19	0.2068	−0.3543	−4.3660
Rostralanteriorcingulate_l	Flight22-control22	0.1130	−0.4458	−5.4940
Rostralanteriorcingulate_l	Flight22-flight19	0.0000	−0.1260	1.5520
Rostralanteriorcingulate_l	Control22-control19	0.0453	−0.0344	0.4240

l, left hemisphere.

### 3.4 Analysis of correlation

No significant correlation was found.

## 4 Discussion

In the current study, we investigated the effects of flight training on the cortical characteristics of flying cadets. The flight group exhibited thinner cortical thickness in left superiorparietal, left supramarginal and right entorhinal area than 3 years before. Meanwhile, the control group either remained unchanged or presented an opposite trend of change. At the second visit, the flight group demonstrated a greater cortical surface area in many brain regions. Concurrently, the control group exhibited a reduced cortical surface area in these regions compared to the first visit. A more significant difference was that, in the left rostral anterior cingulate area, the flight group presented prominent gyri, while the control group showed sunken sulci. Moreover, after 3 years, this difference became increasingly larger.

All participants in the study were enrolled at CAFUC, the nation’s sole full-time higher education institution dedicated to civil aviation pilot training. The ab initio training program at CAFUC comprises two distinct phases: 2 years of ground-based theoretical instruction followed by approximately 2 years of practical training. This hands-on training encompasses both flight simulator sessions and actual airborne operations. In contrast, students in the control group exclusively completed 4 years of classroom-based theoretical curriculum without participating in any flight training modules.

In the human brain, cortical surface topology serves as a predictor of underlying functional representations. Cortical thickness is determined by cellular density, soma size, synaptic connections, and myelination extent (Eickhoff et al., 2005). Cortical surface area, conversely, correlates with the number of cortical columns - modular units exhibiting neuroplasticity. Notably, primate evolution demonstrates rapid expansion of cortical surface area with minimal changes in cortical thickness (Rakic, 1988, 1995). In this study, the flight group exhibited cortical thinning in the left superior parietal lobule, left supramarginal gyrus, and right entorhinal cortex. These regions are functionally critical: The supramarginal gyrus integrates multisensory inputs for vestibular processing (Kheradmand et al., 2015), while the superior parietal lobule facilitates sensorimotor integration for body schema maintenance (Wolpert et al., 1998). The entorhinal cortex, anatomically adjacent to the orbitofrontal cortex, relays sensory-affective information to the latter region which encodes stimulus valence (Sakurai et al., 2015). Contrary to the intuitive assumption that thicker cortex indicates functional superiority, neuroimaging evidence suggests thinner cortex may reflect optimized neural circuitry. fMRI studies demonstrate inverse correlations between cortical thickness and task-related activation intensity (Lu et al., 2009), potentially indicating more efficient information processing in thinner cortices (Liem et al., 2012). Flight operations impose exceptional cognitive demands: Pilots must continuously integrate visual (instrument panels), auditory (radio communications), and vestibular (spatial orientation) inputs to monitor aircraft kinematics and execute appropriate maneuvers. This sustained multisensory integration load possibly drives structural adaptations in associated cortical regions. Longitudinal training effects manifested as structural expansion in primary sensorimotor regions (postcentral and paracentral lobules). These areas mediate tactile feedback and motor execution - skills extensively trained through repetitive manipulation of flight controls (yoke, rudder pedals). Use-dependent cortical reorganization principles suggest that such structural changes may represent experience-dependent neuroplasticity.

Cortical folding is strongly associated with rapid expansion of functionally specialized regions. Studies have revealed systematic organization between sulcal morphology and functional topography (Weiner et al., 2018), with sulcal depth and gyrification index serving as quantifiable markers of cortical folding complexity (Bernardoni et al., 2018). This gyrification process constitutes a lifelong dynamic continuum that demonstrates sensitivity to both neurodevelopmental processes and degenerative changes (Kochunov et al., 2005; Im et al., 2008). Notably, sulcal shallowing has been consistently associated with cortical atrophy in dementia spectrum disorders (Im et al., 2006). In the left rostral anterior-cingulate region (rACC), our flight-trained cohort exhibited predominant gyral configurations, whereas control participants demonstrated sulcal predominance. This neuroanatomical divergence became significantly amplified following extended flight training. The rACC plays a pivotal role in concurrent task management (Dreher and Grafman, 2003), maintaining extensive connectivity with prefrontal, parietal, and subcortical networks implicated in cognitive valuation. Its capacity for real-time multisensory integration establishes the rACC as a critical neural substrate for sustaining continuous behavioral adaptation in dynamic environments (Monosov et al., 2020). Specifically, the rACC demonstrates three signature computational capacities: (1) real-time detection of response conflicts under uncertainty (Carter and van Veen, 2007), (2) dynamic action plan updating via prefrontal-hippocampal circuits (Shenhav et al., 2013), and (3) adaptive switching between proceduralized and effortful processing modes (Botvinick et al., 2004). Aviation operations inherently demand precise coordination of concurrent cognitive-motor tasks. Professional pilots must continuously monitor cockpit instrumentation, execute coordinated limb movements for aircraft control, and process auditory commands from air traffic control – operations routinely assessed through standardized multi-tasking evaluations. Importantly, the flight environment is inherently dynamic, requiring pilots to continuously monitor both internal and external cockpit conditions. They must adapt their operational strategies in real time based on situational demands and dynamically allocate control between automated systems and manual interventions. These complex cognitive functions are closely associated with the rACC. The observed divergent trajectories of rACC morphological changes between experimental groups likely reflect neuroplastic adaptations attributable to sustained flight training.

The present study failed to identify any correlation between flight training duration and cortical metrics. This null finding may be attributable to the standardized training protocol implemented across all flight cadets, resulting in identical flight hours and no inter-individual variation in accumulated flight hours. The sole distinguishing factor was the temporal duration required to complete the equivalent flight training curriculum. Consequently, flight training duration may not constitute a reliable neurobiological marker for assessing training-induced structural brain modifications. In subsequent research, comparative analyses should be conducted among flight trainees with varying flight hours.

The primary limitation of this study lies in its relatively small sample size, which may have impacted the statistical reliability of the findings. Longitudinal tracking of specific populations (e.g., cadets undergoing flight training) presents practical challenges; however, this study represents the first investigation into the effects of flight training on brain structure. As an exploratory study, the final results were not subjected to multiple comparison correction. Consequently, caution should be exercised when interpreting and applying these findings. Nevertheless, as a pioneering investigation, the results provide valuable insights for subsequent research in this field. Of course, the absence of female subjects in this study indeed has a certain influence on the generalizability of the findings. In addition, it should be noted that there were inter-group differences in some indicators of the brain structure of the two groups of subjects at baseline. This may have led to an overestimation of some interaction effects. This problem can be addressed in future studies by increasing the sample size. Furthermore, this study only considered the changes in gray matter structure and lacked data from task-related fMRI, which resulted in insufficient correlation between this study and behavioral performance. Finally, this study only collected data at two time points, which is insufficient to comprehensively and systematically reveal the dynamic changes in the impact of the training process on the brain. Based on this exploratory study, in the future, a large-sample, long-term tracking study will be conducted. During the training period, data will be collected every 6 months, and after the trainees start working, data will be collected once a year. Continuous tracking will be carried out in order to obtain a comprehensive and complete model of the impact of flying on the brain structure.

In conclusion, our analysis of pre- and post-flight training brain structural data revealed significant differences between flight cadets and the ground-based control group. Specifically, the flight group discovered structural changes in brain regions associated with multisensory integration (e.g., the posterior parietal cortex) and multitasking (e.g., the prefrontal cortex). These changes may be related to the brain plasticity caused by flight training. Future research should prioritize longitudinal investigations with larger cohorts to validate these observations, while incorporating functional neuroimaging to elucidate the behavioral relevance of these structural alterations.

## Data Availability

The datasets presented in this study can be found in online repositories. The names of the repository/repositories and accession number(s) can be found in the article/supplementary material.

## Footnotes

^1^ https://surfer.nmr.mgh.harvard.edu/
